# Supplementary material for: ARViS: a bleed-free multi-site automated injection robot for accurate, fast, and dense delivery of virus to mouse and marmoset cerebral cortex
Source: Nat Commun. 2024 Sep 10;15:7633. doi: 10.1038/s41467-024-51986-3 (PMC11387507; doi:10.1038/s41467-024-51986-3)
Supplement: Supplementary file 1 — Supplementary Information [file 41467_2024_51986_MOESM1_ESM.pdf]

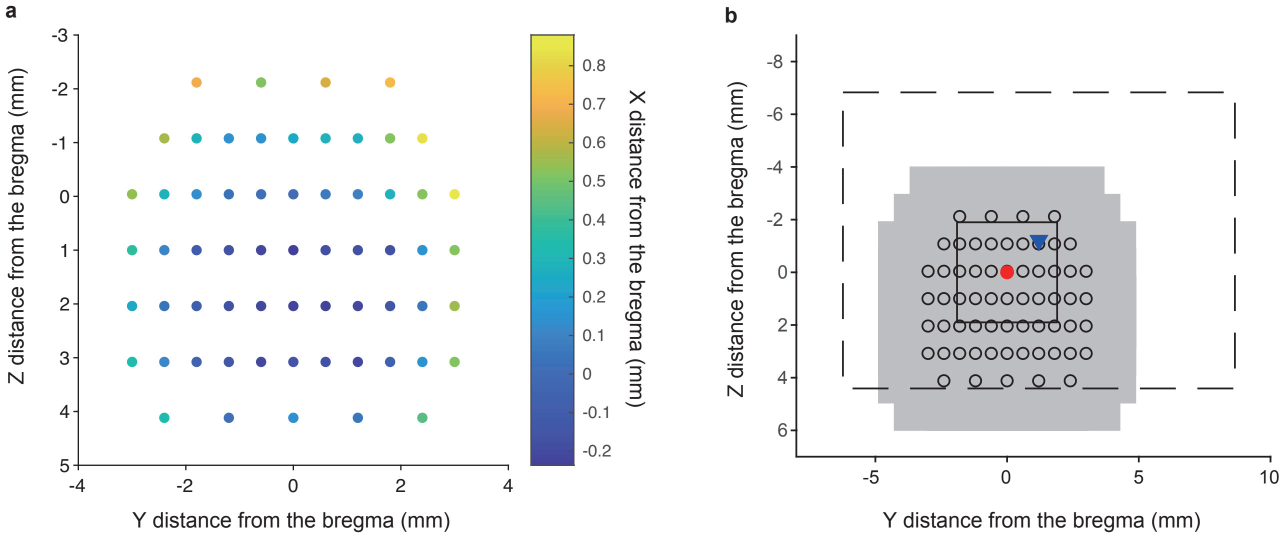

Supplementary Figure 1. Relationship between the image acquisition points and the photographic field of view.
a, Representative plots of the image acquisition points (dots) for mice. Sixty-two points were arranged on the surface measured by the laser distance sensor. The pseudo-colored bar shows the measured X coordinate of each point relative to the bregma. **b**, Schematic diagram illustrating the relationship between image acquisition points and the corresponding frames for SA-UNet inputs. Open circles denote all the image acquisition points shown in (**a**). For an image captured at the position indicated by the red filled circle, the resulting field of view from Camera S is delineated by the dashed frame. The blue triangle marks the center of this field of view. The red filled circle also signifies the pipette tip position within the corresponding field of view of Camera S when the pipette tip was moved down at the focal plane of Camera S. The solid black rectangle was the input image of this field of view for SA-UNet. The gray shading represents the area of the image where all 62 SA-UNet output images were stitched together (original stitched image). Source data are provided as the Source Data file.

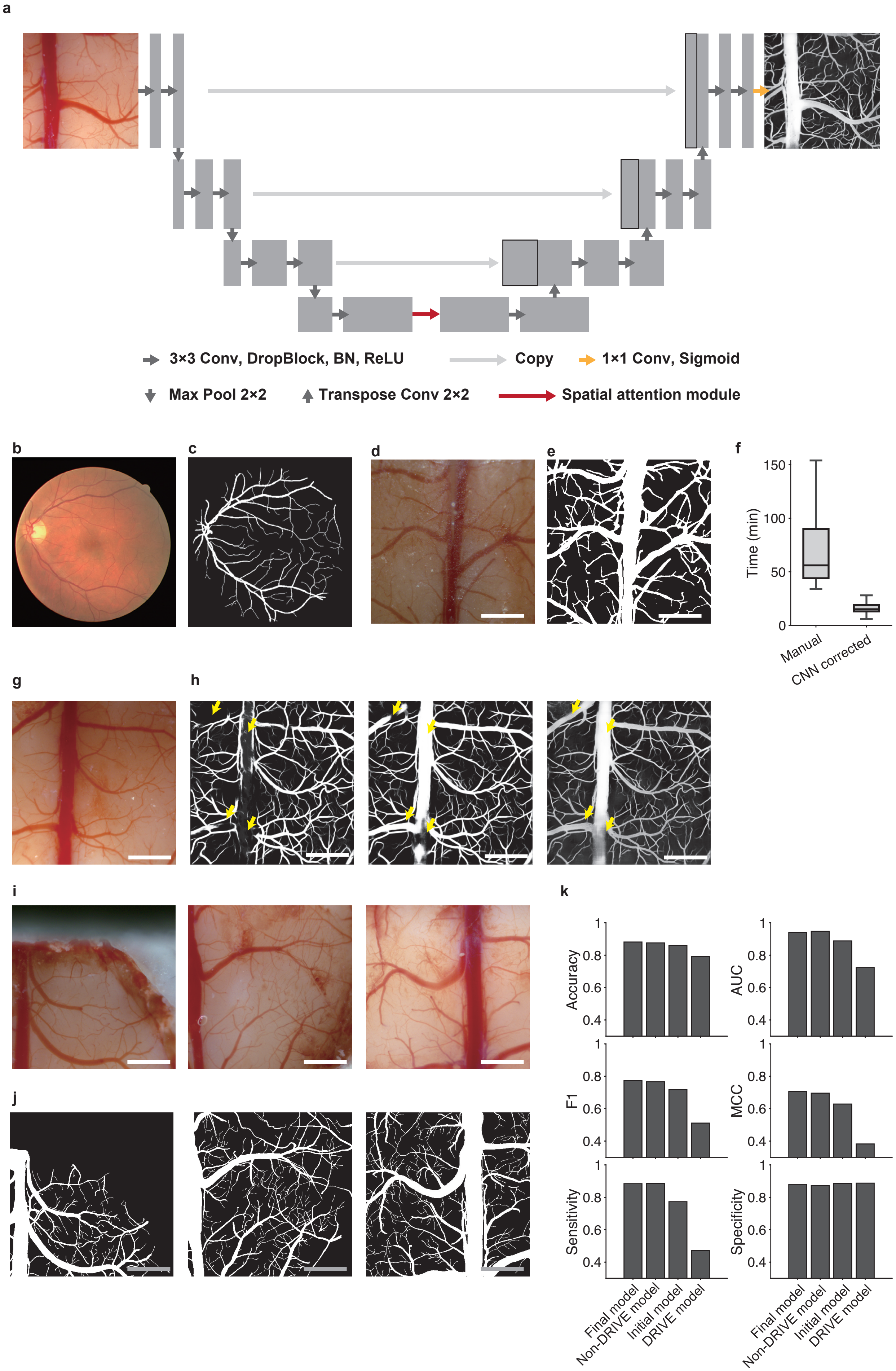

a
3×3 Conv, DropBlock, BN, ReLU
Copy
1×1 Conv, Sigmoid
Max Pool 2×2
Transpose Conv 2×2
Spatial attention module
b
c
d
e
f
Time (min)
150
100
50
0
Manual
CNN corrected
g
h
i
j
k
Accuracy
AUC
F1
MCC
Sensitivity
Specificity
1
0.8
0.6
0.4
Final model
Non-DRIVE model
Initial model
DRIVE model

Supplementary Figure 2. Building the neural network for the vessel segmentation.
a, Schematic of the architecture of SA-UNet (Guo, et al., 2021). SA-UNet is a convolutional neural network originally designed for retinal vessel segmentation. It has two important features: a U-shaped encoder (left side)-decoder (right side) structure that achieves abstraction of spatial information while preserving the resolution, and a spatial attention unit for adaptive feature refinement (middle). The convolutional process includes 3 × 3 convolution, a DropBlock, a batch normalization (BN) layer, and a rectified linear unit (ReLU) (short gray right-pointing arrows). Max pooling with a stride of two handles the downsampling, then feature channels are doubled with each step (downward arrows). Decoder stages include a 2 × 2 transposed convolution, which halves the feature channels, and concatenation with the feature map copied from the encoder layers (upward arrows and long gray right-pointing arrows). The spatial attention module is set between the encoder and the decoder (red right-pointing arrow). In the final layer, a 1×1 convolution and sigmoid activation function is used to obtain the output segmentation map (yellow right-pointing arrow). See the original paper (Guo, et al., 2021) for further information. **b**, Raw retinal fundus vasculature image from the DRIVE dataset with a 45° field of view. **c**, Training label image with vessels denoted by white pixels. The image shown in (**b**) is used. **d**, A representative vasculature image of the mouse cortical surface from the training dataset. Scale bar, 1 mm. **e**, Manual annotation of the vasculature in the image shown in (**d**). Scale bar, 1 mm. **f**, Box chart of the time required to create one annotated image when a raw image was manually annotated (left, n = 10 images), and when a raw image was annotated by the CNN and the CNN-annotation was manually modified (right, n = 30 images). **g**, Another example input image of the mouse cortical surface for SA-UNet. This image included a very thick vessel in the midline. Scale bar, 1 mm. **h**, Example output images of vessel segmentation using SA-UNet trained sololy on the DRIVE dataset (left; DRIVE model), the model trained on the DRIVE dataset and six manually created datasets (middle; the initial model), and the model trained on the DRIVE and 33 original datasets (right; the final model). The input image is the image shown in (**g**). Thick vessels were incompletely segmented when it was trained on only (arrows). Scale bar, 1 mm. **i**, Three input images of the test dataset. Scale bar, 1 mm. **j**, Manually labeled images of the test dataset. Scale bar, 1 mm. **k**, Six metrics of the models trained on different training datasets. Accuracy (the proportion of correctly classified instances): 0.881 for the final model, 0.876 for the non-DRIVE model, 0.860 for the initial model, and 0.792 for the DRIVE model. AUC (area under the ROC curve; a measure of the model's performance across all classification thresholds): 0.941 for the final model, 0.957 for the non-DRIVE model, 0.888 for the initial model, and 0.724 for the DRIVE model. F1 Score (the harmonic mean of precision and recall, balancing both metrics): 0.774 for the final model, 0.767 for the non-DRIVE model, 0.718 for the initial model, and 0.511 for the DRIVE model. MCC (Matthews correlation coefficient, a robust metric for evaluating binary classifications, especially in imbalanced datasets, ranging from 1 for perfect prediction to −1 for complete disagreement): 0.706 for the final model, 0.696 for the non-DRIVE model, 0.628 for the initial model, and 0.382 for the DRIVE model. Sensitivity (recall, the proportion of actual positive instances correctly identified): 0.884 for the final model, 0.885 for the non-DRIVE model, 0.773 for the initial model, and 0.472 for the DRIVE model. Specificity (the proportion of actual negative instances correctly identified): 0.881 for the final model, 0.873 for the non-DRIVE model, 0.886 for the initial model, and 0.888 for the DRIVE model. With sensitivity and specificity, if the number of positive and negative examples is biased in one direction or the other, the poorer performing model will have a higher rate of the biased positive or negative determinations and may produce higher values than the better performing model. In fact, in the current case of classification training with a high percentage of negative examples relative to the total sample, specificity was higher with the initial and DRIVE models, which would not be able to correctly determine positive examples. Thus, sensitivity and specificity were not considered in the evaluation of the segmentation performance. The non-DRIVE model showed the second best AUC, F1, and MCC, with values that were not largely different from those of the final model. In addition, the performance of the DRIVE model was much lower than that of the initial model. Thus, for training the SA-UNet for vessel segmentation on the mouse cortical surface, the contribution of the DRIVE dataset was much smaller than that of the mouse data set. Source data are provided as the Source Data file.

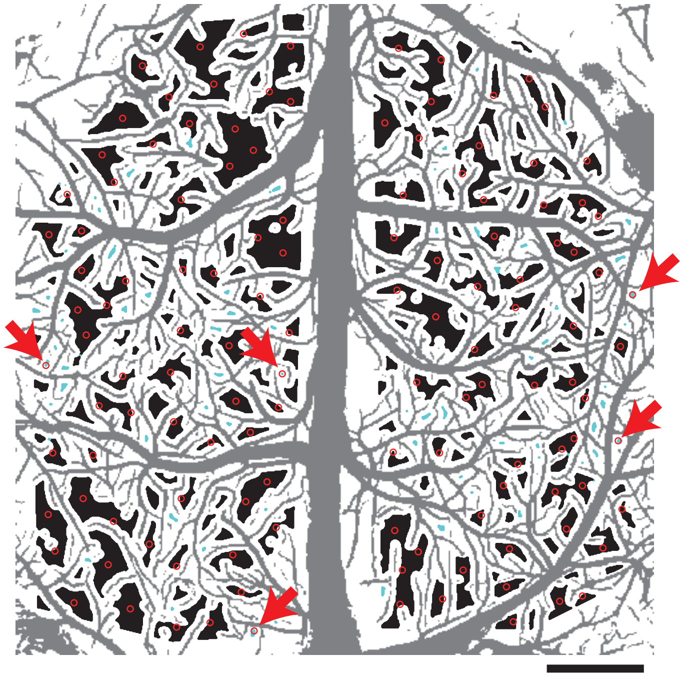

Supplementary Figure 3. Example vessel pattern with safety regions and injection sites.
An example injection site arrangement. The injection sites (red circles) were set in the safety regions and the number of the sites were 127. The safety regions are divided into those with individual areas of more than 30 pixels (black; a pixel size of 7.5 µm) and those with individual areas of 30 pixels or less (cyan). Gray shading illustrates the vessel patterns. White pixels are those which were less than 65 µm from the nearest blood vessel. Although the total area of small spot-like safety regions (cyan) was 753 pixels, and these small regions occupied only 0.92% of the entire area of the safety regions (8.17×10^4 pixels), 3.94% of injection sites (five out of 127 sites) were set in these small regions (red arrows). Scale bar, 1 mm. Source data are provided as the Source Data file.

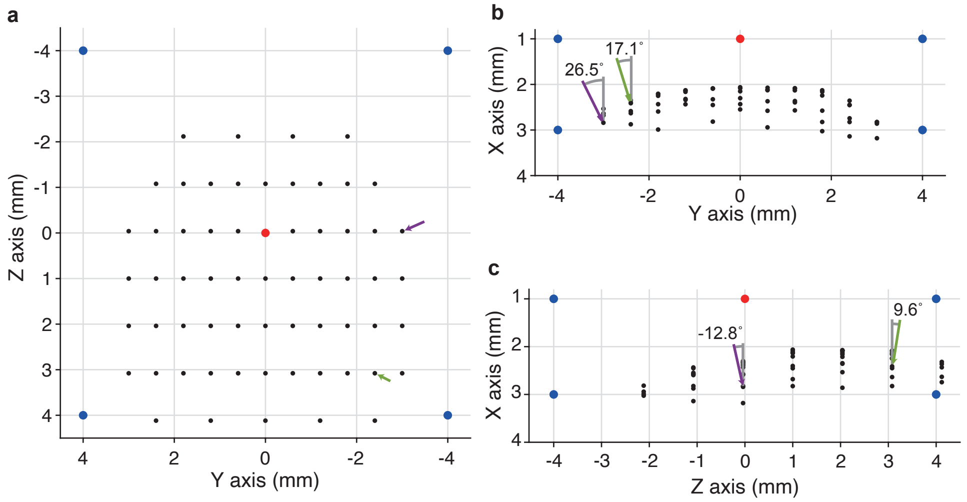

Supplementary Figure 4. Performance of TCP calibration in different coordinates.
a–**c**, Representative Y and Z positions (**a**), X and Y positions (**b**), and Z and X positions (**c**) of the TCP calibration points (red), calibration estimation points (blue), and the points where the cortical surface images were captured (black). Representative XYZ directions perpendicular to the cortical surface are shown by green and purple arrows. Angle values in the W and V rotations for each representative arrow are shown in (**b**) and (**c**), respectively. Source data are provided as the Source Data file.

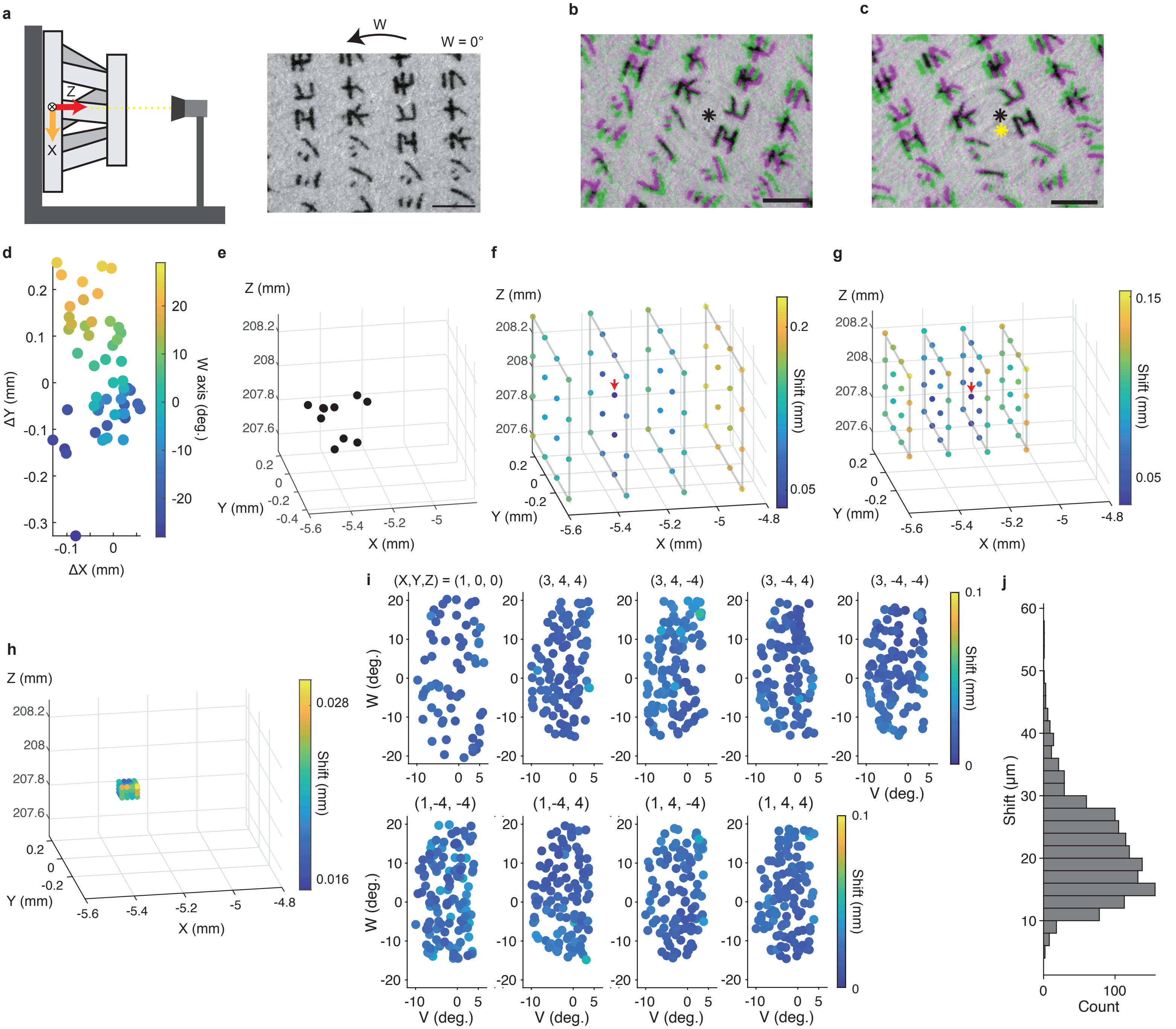

Supplementary Figure 5. Compensation for rotation error.

a, Schematic of the measurement of the shift by the robot stage rotation. Left: For determining the rotation center on the W-axis through multiple image captures, Camera R is positioned in front of the stage. Its optical axis is aligned along the Z axis of the base coordinate system. Right: A picture of a paper with Japanese letters (Katakana) affixed to the robot stage, which was captured by the Camera R when the W rotation was 0 degree. Images at different W rotations were captured, as shown in (**b**,**c**). An arrow indicates the positive direction of the W rotation. Scale bar, 100 mm. **b**, Overlaid images captured at W rotations of –21° (green) and –26° (magenta). The black asterisk indicates the estimated rotation center that was calculated by comparing and matching feature points across the images. Scale bar, 1 mm. **c**, Overlaid images captured at W rotations of 26° (green) and 21° (magenta). The yellow asterisk indicates the estimated rotation center at these rotations. The black asterisk indicates the rotation center estimated from the rotations in (**b**). Scale bar, 1 mm. **d**, XY shift (ΔX and ΔY) of the rotation center against the angle in the W rotation. To generate each data point, an image captured at w degrees in the W rotation was compared with another captured at w + 5 degrees. The rotation center' s coordinates were then calculated according to the procedure outlined in (**a**–**c**). These coordinates were plotted relative to their original positions at 0° in the W rotation. The pseudo-color bar indicates the degree of W rotation (n = 54 images). **e**, The coordinates of $\mathbf{P}^{E}_{\mathrm{Pipette}}$ estimated from the pipette tip movement data, which included the XY shift of the rotation center. Each data point was calculated using measurements taken at different coordinates of the pivot point. **f**, The representative pivot points used for measuring the pipette tip shift during the first grid search. The measurement range was 0.7–0.8 mm, including all estimated coordinates in (**e**). A pseudo-color bar represents the extent of the pipette tip shift. A red arrow indicates the points where the shift was the smallest. **g**, The representative pivot points used for measuring the pipette tip shift during the second grid search. The measurement range was reduced to 0.47 mm, which is two-thirds of the initial range. A pseudo-color bar indicates the length of the pipette tip shift. A red arrow indicates the points where the shift was the smallest. **h**, The representative pivot points used for measuring the pipette tip shift after the final round of grid searching. The measurement range was further narrowed to 0.06 mm. A pseudo-color bar is used to illustrate the extent of the pipette tip shift. **i**, Maps of the pipette tip shift caused by rotation at different positions after applying the

compensation. The coordinates of the measured positions are shown as red and blue dots in Supplementary Figure 4**a**,**b**. **j**, Histogram of the pipette tip shift caused by rotations at the different positions shown in (**i**) (n = 1274). Source data are provided as the Source Data file.

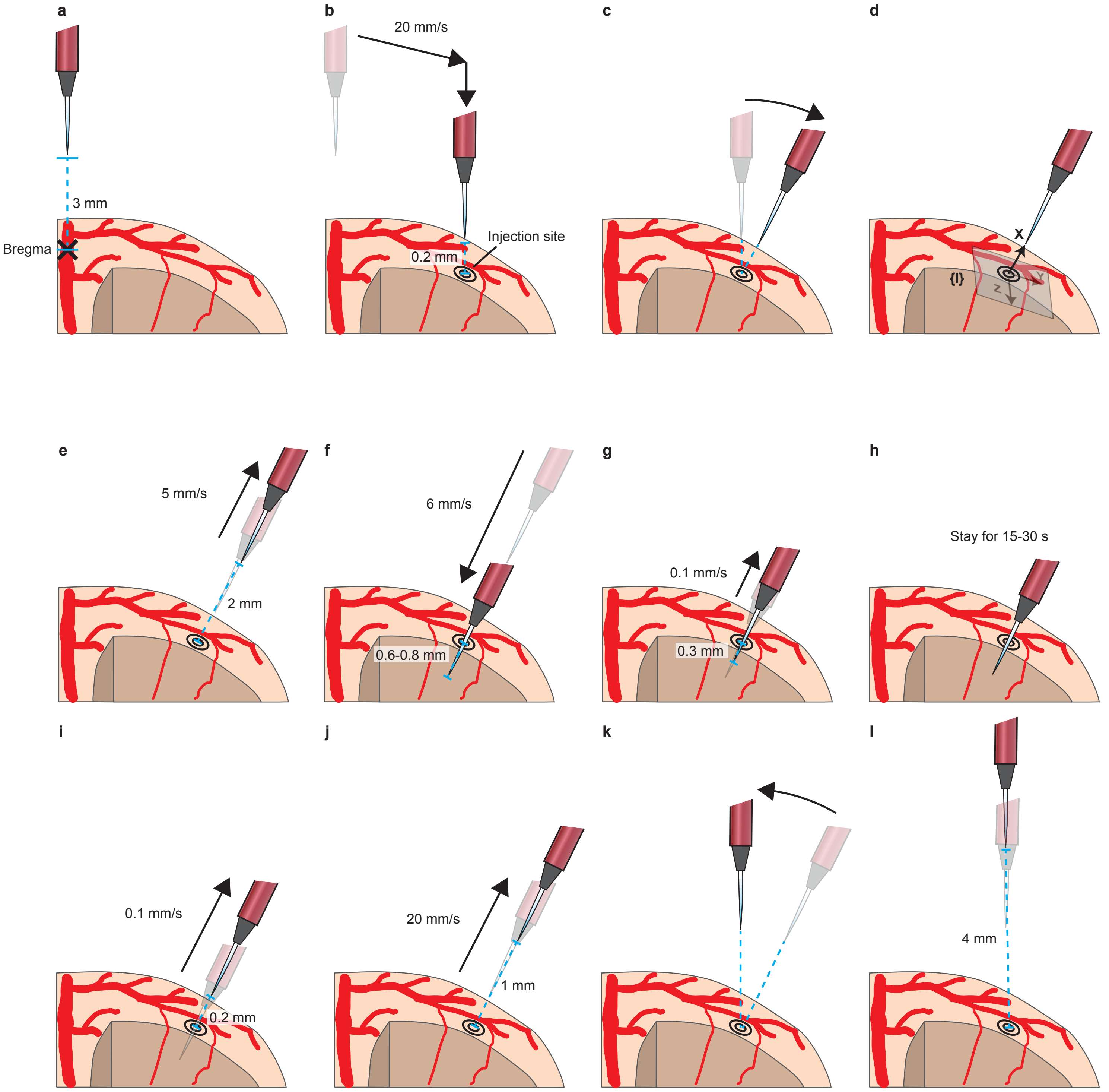

Supplementary Figure 6. Sequential injections into the target injection sites.
The series of pipette tip movements were made according to the following steps. **a**, First, the pipette tip was set 3 mm above the bregma. **b**, It was moved to 1 mm above the first target injection point and then vertically approached to 0.2 mm above the target injection point on the cortical surface at a speed of 20 mm/s. **c**, The angle of the pipette was changed to be perpendicular to the surface. **d**, A new coordinate system with the injection point on the cortical surface as the origin was set as frame {I}. The X axis of the device was aligned with the vertical line to the cortical surface. **e**, The pipette tip was pulled 2 mm back along the X axis in frame {I}. **f**, It was then inserted to a depth of 0.6–0.8 mm from the cortical surface at the injection point. **g**, It was pulled back to the target depth (0.3 mm) at 0.1 mm/s. **h**, The pipette was held for 15–30 s before the injection, then the solution was injected at a speed of 2 nl/s for 15 s. The pipette was held in place for 30 s. **i**, The pipette was pulled back 0.2 mm above the surface along the X axis in frame {I} at a speed of 0.1 mm/s. **j**, The pipette was pulled back 1 mm along the X axis in frame {I} at a speed of 20 mm/s. **k**, The angle was aligned to the initial state. **l**, The pipette was moved to 4 mm above the injection point. Source data are provided as the Source Data file.

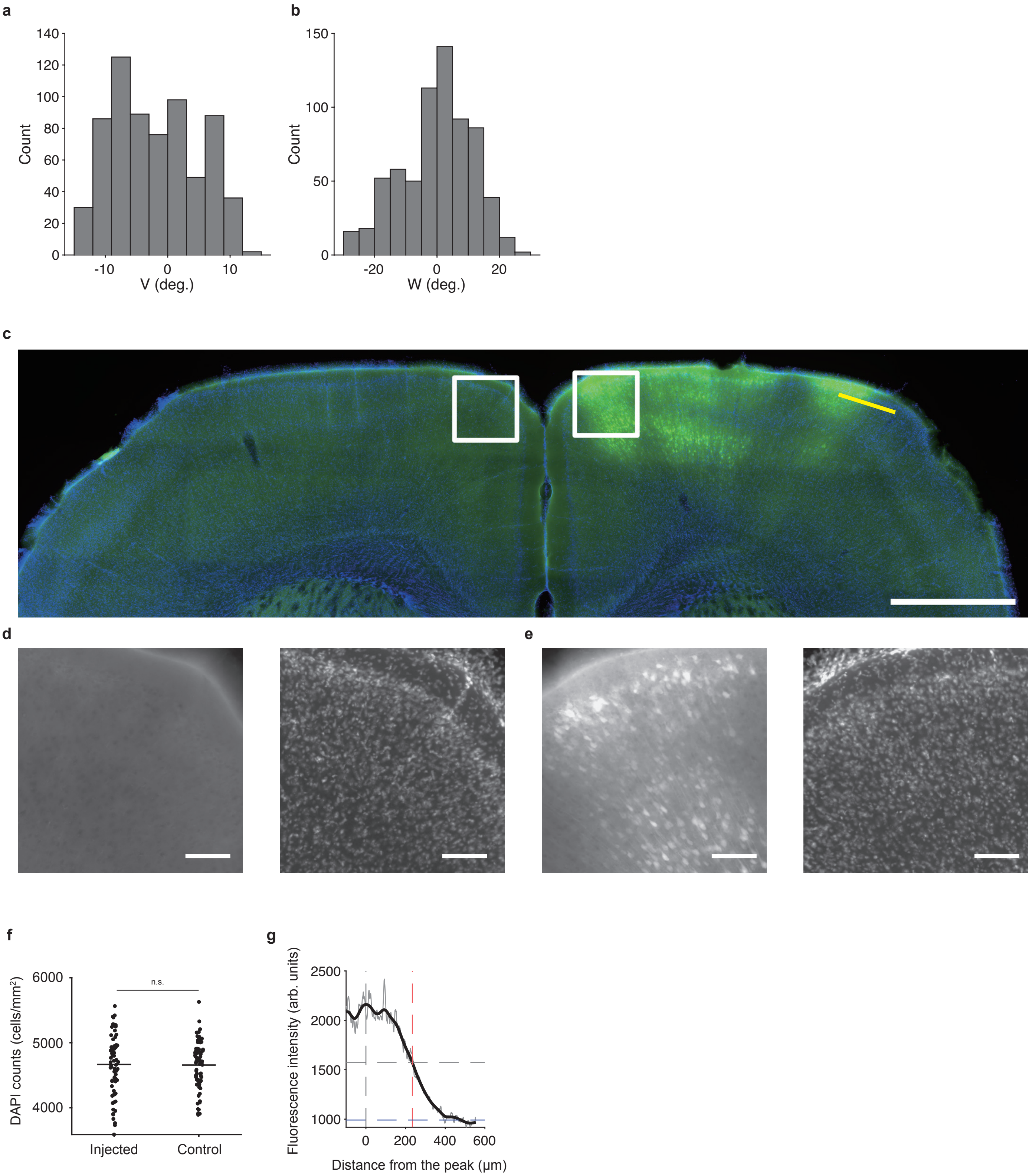

Supplementary Figure 7. Pipette insertion angles and evaluation of cell number loss due to multiple injections in mouse experiments. **a**,**b**, Histogram of the angles in V (**a**) and W (**b**) rotations for the pipette injection into the mouse dorsal cortex (n = 679 from seven mice). **c**, A section of a mouse brain with multiple injections of AAV-hSyn-jGCaMP7c into only the right hemisphere. The white bounding boxes indicate the areas shown in panels (**d**,**e**). Scale bar, 1 mm. **d**,**e**, Magnified images of the left (**d**) and right (**e**) hemispheres with jGCaMP7c signal (left) and DAPI signal (right). **f**, Quantification of DAPI counts in the AAV-injected hemisphere versus the control hemisphere. There was no significant difference between the two groups in a paired *t*-test (two-sided, p = 0.89, n = 73 slices from two mice). **g**, The raw fluorescence signal (gray) and Gaussian-smoothed signal (black) of jGCaMP7c along the yellow line shown in (**c**). The blue horizontal line represents the baseline fluorescence, calculated as the 7th percentile value of the fluorescence in the measured data. The half-decay fluorescence value (horizontal dashed gray line) is the average of the peak value and the baseline. The half-decay distance (red dashed line; 234.22 μm) was measured at the points where the smoothed signal fell below the half-decay fluorescence value. Source data are provided as the Source Data file.

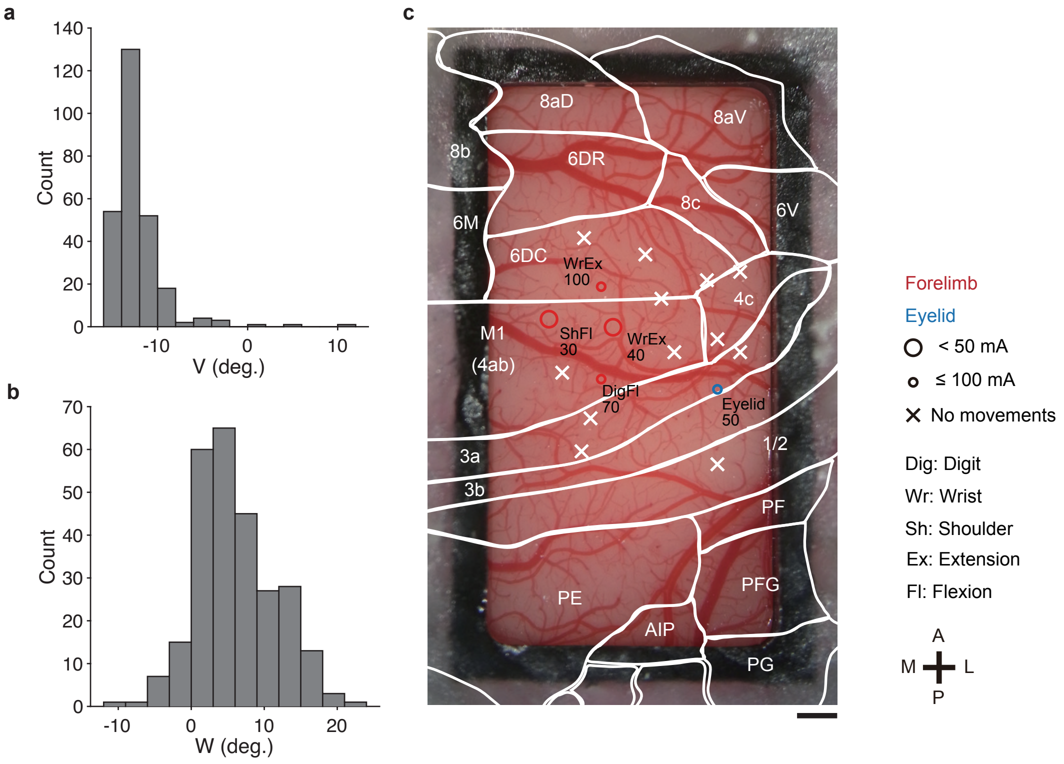

Supplementary Figure 8. Pipette insertion angles and intracortical microsimulation in the marmoset.
a,**b**, Histogram of the angles in V (**a**) and W (**b**) rotations for the pipette injection into the marmoset frontoparietal cortex (n = 266 from one marmoset). **c**, The results of ICMS of the sensorimotor cortex in the marmoset. Red circles indicate stimulation sites where forelimb movements were observed, and the blue circle represents a site where eyelid movement was observed. The diameters of the circles represent the minimal amplitude of the stimulation current that the movements were evoked by. Crosses indicate sites where no movements were observed. A, P, M, and L stand for anterior, posterior, medial, and lateral, respectively. Scale bar, 1 mm. Source data are provided as the Source Data file.

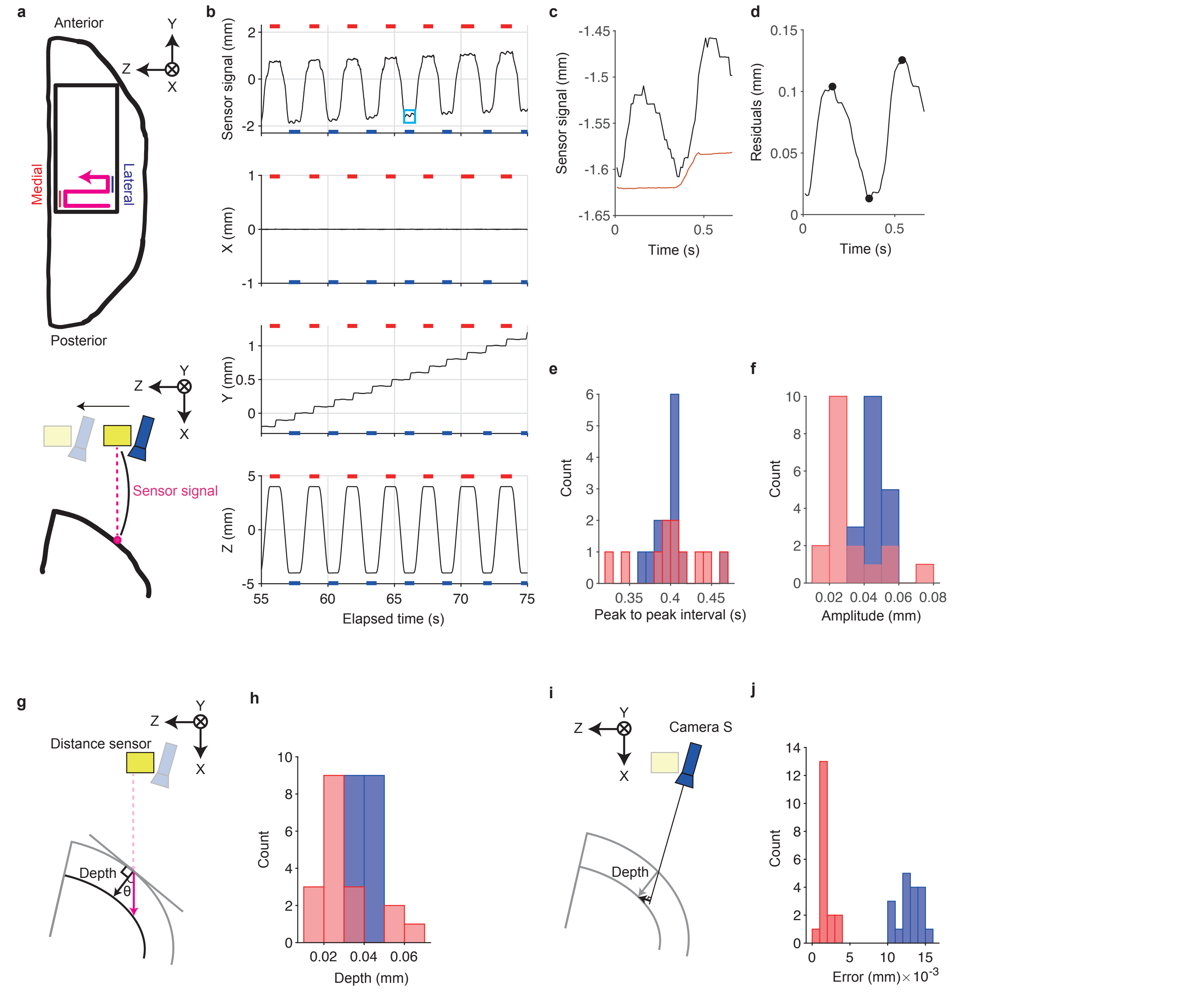

Supplementary Figure 9. Estimation of brain pulsation of the marmoset.
a, Schematic representation of the method used to measure the surface of the marmoset brain. Top, a part of the trajectory of YZ movement of the laser distance sensor (magenta) viewed from the X (depth) axis. The depth fluctuation due to the animal movement was estimated in the period without laser distance sensor movement along the Z axis (red line on the medial side and blue line on the lateral side). Bottom, laser distance sensor movement viewed from the Y (anterior-to-posterior) axis. Camera S (blue) is positioned at approximately 16 degrees tilt relative to the optical axis of the laser distance sensor (yellow; equal to X axis). From the laser distance sensor signal, the depths of individual points on the cortical surface in the X direction were measured. The marmoset was tilted at an angle of approximately 13 degrees to the Z axis so that the cortical surface within the craniotomy was as close to perpendicular to the optical axis of camera S as possible. **b**, Top, representative time course of the laser distance sensor signal, shown along with the corresponding time courses of positions of the laser distance sensor in the X (second top), Y (third top), and Z (bottom) axes. Periods with no Z direction movement are shown in red (medial side) and blue (lateral side). The time segments shown in red and blue were used to isolate these fluctuations because, in the other periods when the sensor moved fast in the Z direction, depth fluctuations caused by animal movements were masked by depth changes of the cortical surface scanning in the Z-axis direction (ranging from –4 to 4 mm), which were nearly a hundred times larger. **c**, Example laser distance sensor signal (black line) shown in the cyan box in (**b**), and corresponding linear regression curve explained by the position of the laser distance sensor (orange line). **d**, Smoothed residual signal of the linear regression curve subtracted from the raw data (black line). The peaks and troughs are indicated by black circles. **e**, Distribution of peak-to-peak interval (red, medial side; blue, lateral side). **f**, Distribution of the amplitude of waves (red, medial side; blue, lateral side). **g**, Schematic illustrating calculation of the deviation perpendicular to the cortical surface (black arrow) from the deviation in the X direction measured by the laser distance sensor (magenta arrow). The black curve represents the brain surface, while the gray one shows the deviated surface with pulsation. Assuming the brain surface curvature is negligible, the perpendicular pulsation component can be approximated by multiplying the laser-measured vector by the cosine of the angle between the perpendicular vector of the surface and the laser trajectory (θ), which was 12.9 and 30 degrees in the medial and lateral sides, respectively. **h**, Distribution of deviation perpendicular to the cortical surface (red, medial side; blue, lateral side). **i**, Schematic illustrating estimation of the deviation in a plane parallel to the cortical surface in the Camera S measurement (black arrow). **j**, Distribution of the estimated deviation in Camera S measurement; red, medial side; blue, lateral side. Source data are provided as the Source Data file.

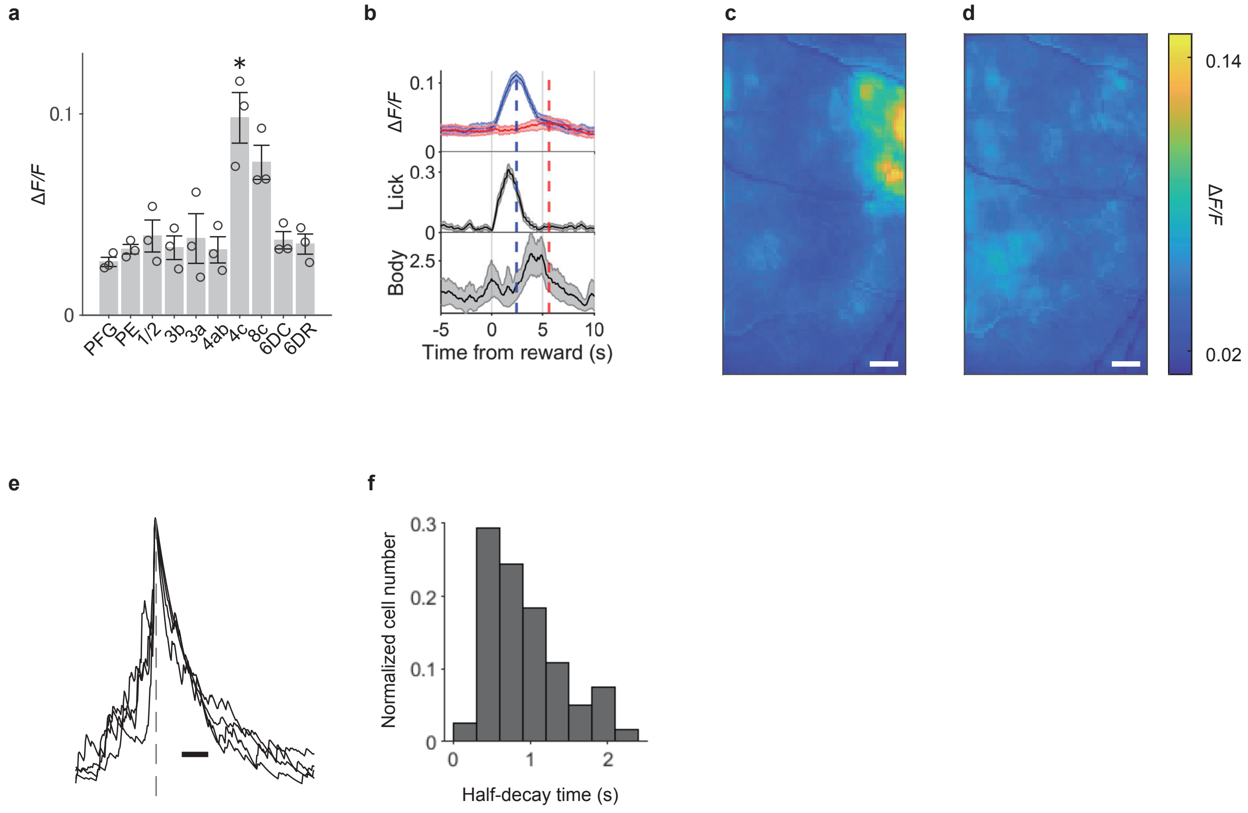

Supplementary Figure 10. Fluorescence signals of different cortical areas around reward timing and half-decay time of Ca^{2+} signal in the marmoset.

a, Averaged Δ*F*/*F* values for the ten cortical areas between 1–3 s after the reward onset for individual sessions, accompanied by a bar plot representing the average value across sessions (error bar denotes the standard error). A one-way ANOVA revealed a significant difference among areas (n = 3 sessions, $F_{10,22} = 8.41$, **p = 1.6 × 10^{-5}). Based on the assumption that area 4ab demonstrated the strongest response to body movement, and that any cortical areas with a greater response might respond to something other than body movement, a one-sided Student' s *t*-test with Bonferroni correction was conducted with area 4ab against other areas to investigate whether this was true. Area 4c exhibited significantly larger responses than area 4ab. Data are plotted only for significant results using a Bonferroni correction threshold of 0.05/9 (n = 3 sessions, *p = 0.0049 < 0.0056). **b**, Δ*F*/*F* signals aligned to the reward delivery timing in area 4c (blue) and area 4ab (red), lick frequency, and body movements. Shading indicates ± standard error (n = 3 sessions). Dashed lines indicate the timing of the peak of Δ*F*/*F* signals in area 4c (blue, 2.4 s) and area 4ab (red, 5.6 s). **c**,**d**, Cortical maps of the averaged Δ*F*/*F* signals 2.4 s (**c**) and 5.6 s (**d**) after the reward delivery. Scale bar, 1 mm. **e**, Four representative traces of calcium transients that were obtained with two-photon imaging of the marmoset motor cortex. The traces were aligned to the peak timing and the amplitude was normalized to the peak amplitude. Scale bar, 1 s. **f**, Histogram of the half decay time constant (0.94 ± 0.05 s; n = 341 ROIs from 4 sessions). To evaluate the half-decay time of the calcium transients, we calculated the half-decay time of calcium transients with peaks more than four standard deviations of the Δ*F*/*F* traces that had no other peak from 3 s before to 6 s after the peak timepoint when averaged over each imaging session in each of all the detected ROIs. To calculate the half-decay time of bursts with fewer than five spikes, the calcium transients with peak values of less than 200% were subjected to analysis (129 ROIs) (Chen et al., Nature 499, 295-300, 2013). The half-decay value was calculated as the average of the baseline (averaged for 3–2.5 s before the peak timing) and the peak value. The half-decay time was measured at the points where the signal fell below the half-decay value. Source data are provided as the Source Data file.

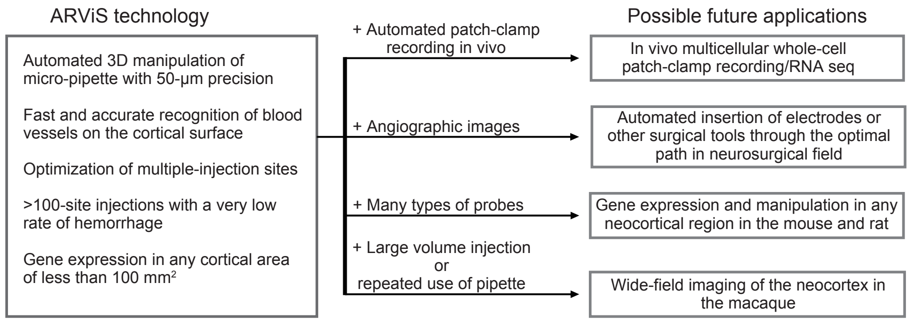

Supplementary Figure 11. Summary of ARViS advantages and possible future applications of ARViS.

Supplementary Table 1. Experimental schedule

Robot process	Time (min)	Related scripts	Animal surgery	Time (min)
Pipette preparation and camera focusing	5	main.m		
Estimating rotation of the camera and the robot	5	TCPCalibration.m	Craniotomy of the animal	80–100
Estimating the coordinates of the needle tip	20			
Measuring needle dislocation due to rotation	70			
Scanning the brain surface	5	ScanSurface.m		
Segmentation with SA-UNet	1	SA-UNet		
Pixel classifier training for realtime closed-loop evaluation of vessels	1	IntegrateSurface.m		
Projecting the vessel segmentation onto the 3D surface	5	Autostitch, IntegrateSurface.m		
Defining the area of injections manually	1	main.m		
Calculating appropriate arrangement of injection sites	5			
Virus loading	1–5			
Automated Injection	1 per site			
			Implanting glass cranial window	5

Supplementary Table 2. Comparison between ARViS and existing methods.

	ARViS	Moccia et al. (Ref. 53)	Rosa® (Ref. 32,33)	NeuroMate (Ref. 55)	iSYS1 (Ref. 56)
Accuracy	50 μm	N/A	760 μm	860 μm	900 μm
Safety margin	65 μm	2000 μm	N/A	N/A	N/A
Diameter of electrode/pipette	30 μm	N/A	Not reported	Not reported	2.2 mm
Automation	Yes	No	Yes	Yes	No
Vessel detection	Yes	Yes	Yes	Yes	No
Multiple injections	Yes	N/A	No	No	No
Target organism	Mouse & Marmoset	Cray phantom	Human	Human	Human
