# Supplementary material for: ARViS: a bleed-free multi-site automated injection robot for accurate, fast, and dense delivery of virus to mouse and marmoset cerebral cortex
Source: Nat Commun. 2024 Sep 10;15:7633. doi: 10.1038/s41467-024-51986-3 (PMC11387507; doi:10.1038/s41467-024-51986-3)
Supplement: Supplementary file 3 — Description of additional supplementary files [file 41467_2024_51986_MOESM3_ESM.pdf]

Description of additional Supplementary Files

Supplementary Movie 1 - V-W rotation of the pipette captured by Camera L and Camera R after all calibration
