# Supplementary material for: ARViS: a bleed-free multi-site automated injection robot for accurate, fast, and dense delivery of virus to mouse and marmoset cerebral cortex
Source: Nat Commun. 2024 Sep 10;15:7633. doi: 10.1038/s41467-024-51986-3 (PMC11387507; doi:10.1038/s41467-024-51986-3)
Supplement: Supplementary file 5 — Reporting Summary [file 41467_2024_51986_MOESM5_ESM.pdf]

nature portfolio

Corresponding author(s): Masanori Matsuzaki

Last updated by author(s): Aug 8, 2024

Reporting Summary

Nature Portfolio wishes to improve the reproducibility of the work that we publish. This form provides structure for consistency and transparency in reporting. For further information on Nature Portfolio policies, see our Editorial Policies and the Editorial Policy Checklist.

Statistics

For all statistical analyses, confirm that the following items are present in the figure legend, table legend, main text, or Methods section.

n/a	Confirmed	
☐	☒	The exact sample size (*n*) for each experimental group/condition, given as a discrete number and unit of measurement
☐	☒	A statement on whether measurements were taken from distinct samples or whether the same sample was measured repeatedly
☐	☒	The statistical test(s) used AND whether they are one- or two-sided<br>*Only common tests should be described solely by name; describe more complex techniques in the Methods section.*
☐	☒	A description of all covariates tested
☐	☒	A description of any assumptions or corrections, such as tests of normality and adjustment for multiple comparisons
☐	☒	A full description of the statistical parameters including central tendency (e.g. means) or other basic estimates (e.g. regression coefficient) AND variation (e.g. standard deviation) or associated estimates of uncertainty (e.g. confidence intervals)
☐	☒	For null hypothesis testing, the test statistic (e.g. F, t, r) with confidence intervals, effect sizes, degrees of freedom and P value noted<br>*Give P values as exact values whenever suitable.*
☒	☐	For Bayesian analysis, information on the choice of priors and Markov chain Monte Carlo settings
☒	☐	For hierarchical and complex designs, identification of the appropriate level for tests and full reporting of outcomes
☒	☐	Estimates of effect sizes (e.g. Cohen's *d*, Pearson's *r*), indicating how they were calculated

Our web collection on statistics for biologists contains articles on many of the points above.

Software and code

Policy information about availability of computer code

Data collection	One-photon imaging was conducted using a fluorescent zoom macroscope equipped with a high- numerical aperture macro lens (PlanNEOFLUAR Z 1.0×, NA 0.25; Carl-Zeiss, Germany). An EM-CCD camera with 512 × 512 pixels (iXon Ultra 888; Andor Technology, UK) was used as photodetector. The center wavelength of the LED for the wide-field calcium imaging was set to 470 nm, and the light intensity under the device was 6 mW. Two-photon imaging was conducted with a custom-built two-photon microscopy (Olympus) system equipped with a water immersion objective lens (Olympus XLPLN10XSVMP, NA 0.6; working distance 8 mm) and an Nd-based fiber-delivered femtosecond laser (Femtolite FD/J-FD-500, pulse width of 191–194 fs, repetition rate of 51 MHz; IMRA, USA) at a wavelength of 920 nm.
Data analysis	All calcium imaging data was processed using MATLAB (R2021a, R2022a; MathWorks, USA), and ImageJ (National Institute of Health, USA). NoRMCorre (https://github.com/flatironinstitute/NoRMCorre) was used for the analysis.

For manuscripts utilizing custom algorithms or software that are central to the research but not yet described in published literature, software must be made available to editors and reviewers. We strongly encourage code deposition in a community repository (e.g. GitHub). See the Nature Portfolio guidelines for submitting code & software for further information.

Data

Policy information about availability of data

All manuscripts must include a data availability statement. This statement should provide the following information, where applicable:

- Accession codes, unique identifiers, or web links for publicly available datasets
- A description of any restrictions on data availability
- For clinical datasets or third party data, please ensure that the statement adheres to our policy

All data supporting the findings of this study are available within the article and its supplementary files. Any additional requests for information can be directed to, and will be fulfilled by, the corresponding authors. Source data are provided with this paper. The imaging data generated in this study for training SA-UNet are deposited in the GitHub under accession code DOI: 10.5281/zenodo.13220760 https://github.com/nomurshin/ARViS_Automated_Robotic_Virus_injection_System/tree/main/Data/Final_model_dataset. All computer codes to realize the ARViS have been deposited in the GitHub under accession code DOI: 10.5281/zenodo.13220760 https://github.com/nomurshin/ARViS_Automated_Robotic_Virus_injection_System.

Research involving human participants, their data, or biological material

Policy information about studies with human participants or human data. See also policy information about sex, gender (identity/presentation), and sexual orientation and race, ethnicity and racism.

Reporting on sex and gender	*Use the terms sex (biological attribute) and gender (shaped by social and cultural circumstances) carefully in order to avoid confusing both terms. Indicate if findings apply to only one sex or gender; describe whether sex and gender were considered in study design; whether sex and/or gender was determined based on self-reporting or assigned and methods used. Provide in the source data disaggregated sex and gender data, where this information has been collected, and if consent has been obtained for sharing of individual-level data; provide overall numbers in this Reporting Summary. Please state if this information has not been collected. Report sex- and gender-based analyses where performed, justify reasons for lack of sex- and gender-based analysis.*
Reporting on race, ethnicity, or other socially relevant groupings	*Please specify the socially constructed or socially relevant categorization variable(s) used in your manuscript and explain why they were used. Please note that such variables should not be used as proxies for other socially constructed/relevant variables (for example, race or ethnicity should not be used as a proxy for socioeconomic status). Provide clear definitions of the relevant terms used, how they were provided (by the participants/respondents, the researchers, or third parties), and the method(s) used to classify people into the different categories (e.g. self-report, census or administrative data, social media data, etc.) Please provide details about how you controlled for confounding variables in your analyses.*
Population characteristics	*Describe the covariate-relevant population characteristics of the human research participants (e.g. age, genotypic information, past and current diagnosis and treatment categories). If you filled out the behavioural & social sciences study design questions and have nothing to add here, write "See above."*
Recruitment	*Describe how participants were recruited. Outline any potential self-selection bias or other biases that may be present and how these are likely to impact results.*
Ethics oversight	*Identify the organization(s) that approved the study protocol.*

Note that full information on the approval of the study protocol must also be provided in the manuscript.

Field-specific reporting

Please select the one below that is the best fit for your research. If you are not sure, read the appropriate sections before making your selection.

☒ Life sciences ☐ Behavioural & social sciences ☐ Ecological, evolutionary & environmental sciences

For a reference copy of the document with all sections, see nature.com/documents/nr-reporting-summary-flat.pdf

Life sciences study design

All studies must disclose on these points even when the disclosure is negative.

Sample size	We did not employ statistical methods to predetermine the sample sizes; however, sample sizes were estimated according to previous methodically comparable laboratory experiments and are similar to those generally employed in the field. We have also added this text in the Methods "Statistics and Reproducibility" section.
Data exclusions	No data was excluded from the analysis.
Replication	In the mice experiment, we performed multiple injections for bleeding evaluation (n = 679 sites from seven mice) and measured accuracy of injections from histological samples (n = 32 sites from one mice). In the marmoset experiment, we obtained multiple one-photon imaging data (n = 3 sessions from one animal) and two-photon imaging data (n = 341 ROIs for neuronal somata from 4 sessions). Reproducibility is ensured by using a sufficient number of injections for evaluating robotic accuracy.

Randomization	The mice were not divided into multiple experimental conditions and the number of the marmoset was one. Thus, the blinded allocation of the animals was not applicable.
Blinding	Blinding was not performed in this study.

Reporting for specific materials, systems and methods

We require information from authors about some types of materials, experimental systems and methods used in many studies. Here, indicate whether each material, system or method listed is relevant to your study. If you are not sure if a list item applies to your research, read the appropriate section before selecting a response.

Materials & experimental systems

n/a	Involved in the study
☒	☐ Antibodies
☐	☒ Eukaryotic cell lines
☒	☐ Palaeontology and archaeology
☐	☒ Animals and other organisms
☒	☐ Clinical data
☒	☐ Dual use research of concern
☒	☐ Plants

Methods

n/a	Involved in the study
☒	☐ ChIP-seq
☒	☐ Flow cytometry
☒	☐ MRI-based neuroimaging

Eukaryotic cell lines

Policy information about cell lines and Sex and Gender in Research

Cell line source(s)	AAV-293 cells are an HEK293 cell line optimized for packaging of AAV virions in the helper-free system (Agilent Technologies, CA, USA). The HEK293 cell line was established from primary embryonic human kidney.
Authentication	Agilent Technologies, CA, USA.
Mycoplasma contamination	Not tested, but originally tested by Agilent Technologies.
Commonly misidentified lines (See ICLAC register)	Not applicable.

Animals and other research organisms

Policy information about studies involving animals; ARRIVE guidelines recommended for reporting animal research, and Sex and Gender in Research

Laboratory animals	This study used one laboratory-bred common marmoset (Callithrix jacchus; female, age 2 year 5 months) and seven C57BL/6 mice (6 males and 1 female, 8–14 weeks old) were used.
Wild animals	This study did not use wild animals.
Reporting on sex	This study used one female marmoset for calcium imaging, seven mice including 6 males and 1 female for bleeding evaluation. We did not conduct sex-based analysis.
Field-collected samples	This study did not use samples collected from the field.
Ethics oversight	All experiments were approved by the Animal Experimental Committee of the University of Tokyo and the Animal Care.

Note that full information on the approval of the study protocol must also be provided in the manuscript.

Plants

Seed stocks	*Report on the source of all seed stocks or other plant material used. If applicable, state the seed stock centre and catalogue number. If plant specimens were collected from the field, describe the collection location, date and sampling procedures.*
Novel plant genotypes	*Describe the methods by which all novel plant genotypes were produced. This includes those generated by transgenic approaches, gene editing, chemical/radiation-based mutagenesis and hybridization. For transgenic lines, describe the transformation method, the number of independent lines analyzed and the generation upon which experiments were performed. For gene-edited lines, describe the editor used, the endogenous sequence targeted for editing, the targeting guide RNA sequence (if applicable) and how the editor was applied.*
Authentication	*Describe any authentication procedures for each seed stock used or novel genotype generated. Describe any experiments used to assess the effect of a mutation and, where applicable, how potential secondary effects (e.g. second site T-DNA insertions, mosiacism, off-target gene editing) were examined.*
